# Mass Flux Calculations Show Strong Allochthonous Support of Freshwater Zooplankton Production Is Unlikely

**DOI:** 10.1371/journal.pone.0039508

**Published:** 2012-06-26

**Authors:** Michael T. Brett, George B. Arhonditsis, Sudeep Chandra, Martin J. Kainz

**Affiliations:** 1 Department of Civil and Environment Engineering, University of Washington, Seattle, Washington, United States of America; 2 Ecological Modeling Laboratory, University of Toronto, Toronto, Ontario, Canada; 3 Department of Natural Resources and Environmental Science, University of Nevada, Reno, Nevada, United States of America; 4 WasserCluster Lunz-Biological Station, Donau-Universität Krems, Lunz am See, Austria; Utrecht University, Netherlands

## Abstract

Many studies have concluded terrestrial carbon inputs contribute 20–70% of the carbon supporting zooplankton and fish production in lakes. Conversely, it is also known that terrestrial carbon inputs are of very low nutritional quality and phytoplankton are strongly preferentially utilized by zooplankton. Because of its low quality, substantial terrestrial support of zooplankton production in lakes is only conceivable when terrigenous organic matter inputs are much larger than algal production. We conducted a quantitative analysis of terrestrial carbon mass influx and algal primary production estimates for oligo/mesotrophic lakes (*i.e.,* TP≤20 µg L^−1^). In keeping with the principle of mass conservation, only the flux of terrestrial carbon retained within lakes can be utilized by zooplankton. Our field data compilation showed the median (inter-quartile range) terrestrial particulate organic carbon (t-POC), available dissolved organic carbon (t-DOC) inputs, and in-lake bacterial and algal production were 11 (8–17), 34 (11–78), 74 (37–165), and 253 (115–546) mg C m^−2^ d^−1^, respectively. Despite the widespread view that terrestrial inputs dominate the carbon flux of many lakes, our analysis indicates algal production is a factor 4–7 greater than the available flux of allochthonous basal resources in low productivity lakes. Lakes with high loading of t-DOC also have high hydraulic flushing rates. Because t-DOC is processed, *i.e.,* mineralized or lost to the sediments, in lakes at ≈0.1% d^−1^, in systems with the highest t-DOC inputs (*i.e.,* 1000 mg m^−2^ d^−1^) a median of 98% of the t-DOC flux is advected and therefore is not available to support zooplankton production. Further, advection is the primary fate of t-DOC in lakes with hydraulic retention times <3 years. When taking into account the availability and quality of terrestrial and autochthonous fluxes, this analysis indicates ≈95–99% of aquatic herbivore production is supported by in-lake primary production.

## Introduction

There is considerable interest [1]–[5] and debate [5]–[7] about the role terrestrial organic matter inputs play in supporting the production of invertebrate and fish consumers in lakes. Understanding the sources of basal resources to lake consumers could have significant implications for ecosystem management, fisheries production and predicting greenhouse gas release from lakes. Several studies have concluded that allochthonous inputs subsidize ≈50% of freshwater zooplankton production in many freshwater systems [1]–[5]. Several researchers [8] have concluded direct terrestrial particulate organic carbon (t-POC) inputs provide 98% of the terrestrial subsidy to zooplankton, while the terrestrially derived dissolved organic carbon (t-DOC) pathway only supports 2%. In contrast, other field studies concluded that the t-DOC → bacterial production → protozoan pathway is the primary route by which terrestrial inputs support zooplankton [2], [4], [9]. Brett et al. [6] pointed out that t-POC is an order of magnitude lower food quality compared to common phytoplankton and therefore very large fluxes from this source would be needed to substantially support zooplankton production. Field determinations have shown t-POC inputs are only ≈1% of phytoplankton production [10] in oligotrophic environments where recent research has suggested allochthonous support of zooplankton is strong, *i.e.,* 20–40% of zooplankton production [5], [11]. It was recently hypothesized that flocculation of t-DOC inputs was the most likely mechanism for large terrestrial subsidies to zooplankton production [5]. The food quality of t-POC derived from flocculating t-DOC is likely to be very low due to the fact that the parent material is extremely recalcitrant to enzymatic attack [12], [13] by bacteria and metazoans, and t-DOC is almost entirely devoid of lipids and proteins necessary for the somatic development of metazoans. Based on the very low quality of terrestrially derived resources, it is widely acknowledged that significant allochthonous support of zooplankton production is only plausible when the flux of terrestrially derived food is considerably larger than the flux of edible algae [5], [6].

In the aquatic ecology literature, it is often stated that the loading of allochthonous organic material to oligotrophic and mesotrophic lakes that can support food web processes is as large or much larger than autochthonous primary production [4], [5], [12]–[15]. However, we are unaware of any quantitative analyses supporting this assertion. It is known that crustacean macrozooplankton are able to consume living or dead particles, therefore t-POC, flocculated t-DOC and bacteria biomass supported by the consumption of t-DOC are possible routes for terrestrial subsidies to freshwater zooplankton production. Bacterial production is supported by both algal exudates [16] and t-DOC [4], and it was recently shown that bacteria production in lakes is strongly associated with (*r^2^* = 0.83) and usually about one-third of phytoplankton production [17]. Further, bacteria are low quality food resources for zooplankton production [18], [19] and are also often too small for many zooplankton to efficiently graze - which necessitates an additional protozoan trophic link and associated energetic loss [9].

The t-DOC flux to lakes is very strongly associated with areal hydraulic loading [20]–[22] because hydraulic inputs vary logarithmically while the t-DOC concentrations associated with these inputs vary arithmetically. Although it is commonly stated the t-DOC present in the water column represents “accumulated” carbon [12]–[14], the fraction of any constituent that persists in the water column actually represents the mass that will be advected [23]. Because lakes with high hydraulic loading rates also usually have short hydraulic retention times (HRTs) advective transport from lakes may be the most common fate of t-DOC in many systems [24]. This is critical for upper trophic levels because only t-DOC that is converted to a particulate form (via assimilation by bacteria or flocculation) can be utilized by zooplankton.

This analysis will quantitatively test the widely held view that the fluxes of terrestrial carbon inputs are “as large to much larger” than pelagic and benthic algal production [4], [5], [12]–[15]. This test is important given that it is generally agreed that it is not possible for terrestrial inputs to make a large contribution to the production of invertebrates and fish in lakes if these inputs do not greatly exceed autochthonous production [5], [6]. This will be done by conducting quantitative analyses of mass flux estimates for several whole lake case studies, including those ecosystems where it has been concluded terrestrial inputs support a large fraction of lake consumer production [3], [5], [8], [10], [11]. The literature reporting fluxes of t-DOC, t-POC, bacterial production, and benthic and pelagic primary production in individual systems will also be analyzed. Furthermore, typical lake hydraulic loading values and t-DOC input concentrations will be used to generate distributions of likely t-DOC loading rates in temperate and boreal lakes. In particular, the importance of t-DOC retention in these mass flux calculations will be considered. Although this study focuses on the role terrestrial organic matter inputs may play in invertebrate and fish production in lakes, and especially lakes with high areal t-DOC loading rates, the conclusions of this study are also highly relevant to the role lakes play as carbon sinks, conduits or sources in the global carbon cycle [25].

## Results and Discussion

### Literature Analysis

Previous studies have concluded that terrestrial organic matter strongly supports zooplankton production in a series of upper Midwestern USA lakes [3], [5], [8], [11], hereafter referred to as the UNDERC lakes. Pace and colleagues assumed terrestrial inputs to these lakes were similar to the concurrent primary production determined for these lakes; *i.e.,* the allochthonous and autochthonous inputs used for their model [8] averaged ≈500 mg C m^−2^ d^−1^, with >80% of the allochthonous inputs in the dissolved phase. Field data collected from UNDERC lakes indicate these terrestrial inputs may be substantially lower. For example, groundwater t-DOC concentrations [26] and areal hydraulic influxes [27] to several UNDERC lakes averaged 12.5±3.8 (± SD) mg L^−1^ and 4.9±3.6 L m^−2^ d^−1^ (or 1.8±1.3 m yr^−1^), respectively, indicating an areal t-DOC influx of 60±43 mg C m^−2^ d^−1^. Calculations based on mean regional rainfall and evapotranspiration [28], and lake watershed to surface area ratios, suggest similar hydraulic loading rates, *i.e.,* 3.8–5.2 L m^−2^ d^−1^, and hence t-DOC loading, to UNDERC lakes in general. UNDERC field data and a meta-analysis [10] for small forest lakes concluded t-POC fluxes averaged 11 mg C m^−2^ d^−1^. Conversely, algal primary production (PPr) averaged 473±60 mg C m^−2^ d^−1^ in Crampton, Paul, Peter and Tuesday lakes [8], [10]. This shows allochthonous inputs to the UNDERC lakes may only be 10–20% of the basal resource flux.

The allochthonous and autochthonous fluxes for the UNDERC lakes are particularly relevant for Crampton Lake due to the detailed direct field determinations of both net phytoplankton production and t-POC influxes available for this system [10]. On the basis of a whole lake ^13^C addition experiment, it was [11] initially concluded that the copepod *Leptodiaptomus minutus* and the cladoceran *Holopedium gibberum* obtained 2% and 31% of their resources from allochthonous sources, respectively. More recently, using a multi-isotope approach, the same authors [5] concluded both *Leptodiaptomus* and *Holopedium* in Crampton obtained ≈30% of their resources from allochthony. The watershed to lake surface area for Crampton is 2.1 [5], Vano et al. [28] reported the mean precipitation and evapotranspiration for this lake district (*i.e.,* 0.85 m yr^−1^ and 58%, respectively), and Christensen et al. [26] reported typical seepage t-DOC concentrations (*i.e.,* 12.5±3.8 mg L^−1^); all of which makes it possible to calculate plausible t-DOC influxes to this lake. According to the data presented in Preston et al. [10], and outlined above, t-POC and t-DOC inputs, and net phytoplankton PPr average 5.2±0.4, 25±8, and 485±49 mg C m^−2^ d^−1^, respectively. That is, terrestrial inputs are only ≈6% of the basal resource flux and 80% as t-DOC. If we assume bacteria have a growth efficiency of ≈10% when metabolizing t-DOC [29], the particulate flux associated with terrestrial inputs would only be 1–2% of net phytoplankton production in Crampton Lake.

### Field Data Compilation

The meta-analysis of individual observations for t-DOC and t-POC inputs to and bacterial and algal production within oligotrophic/mesotrophic lakes also showed that far from being dominant, terrestrial inputs were in general less or much less than autochthonous production. The median (inter-quartile range) measured t-POC, t-DOC, and primary production fluxes were 11 (8–17), 62 (41–100), and 253 (115–546) mg C m^−2^ d^−1^, respectively (Tables S1, S2 and References S1). The terrestrial fluxes averaged 30±22% of the total basal resource flux, and autochthonous production was on average 2.0–3.5 times higher. Bacteria production, median  =  74 (37–165) mg C m^−2^ d^−1^, is more complicated because in lakes this production can be supported by either terrestrial or autochthonous resources [4], [16]. However, the strong statistical association between bacteria and phytoplankton production reported by Fouilland and Mostajir [17], the much lower bacteria growth efficiency on t-DOC than algal exudates [29], and the higher flux of autochthonous than allochthonous carbon indicated by these data are consistent with most bacterial production being supported by in-lake primary production. Further, bacterial production is not large enough to modify the general conclusion that autochthonous production dominates the basal resource flux.

### Scenario Analyses

**Figure 1:**
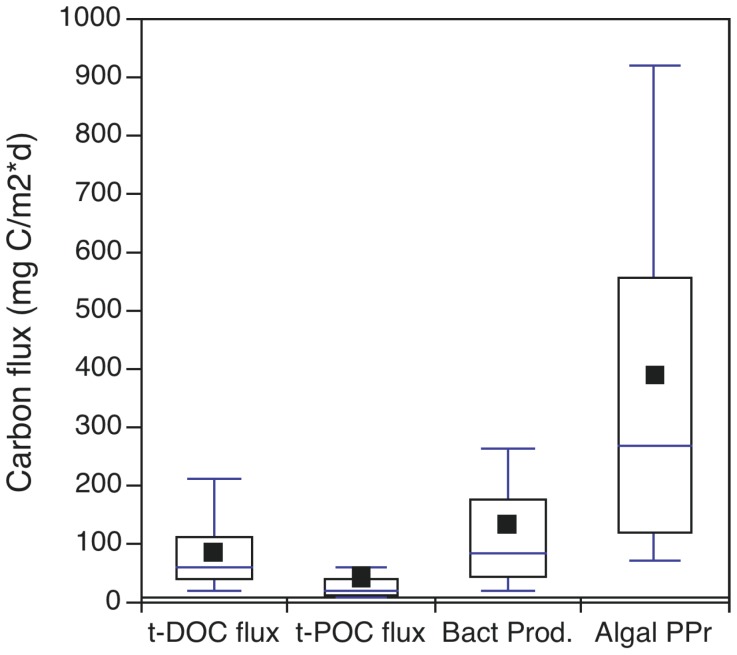
The mass influx of dissolved and particulate carbon from terrestrial sources and the in-lake production of bacteria and benthic/pelagic algae based on individual lake observations. Only data from lakes with total phosphorus <20 µg L^−1^ were used. Terrestrial particulate loading was calculated using the aeolian transport data from Preston et al. [10] while also assuming fluvial t-POC inputs are equal to 10% of t-DOC loading [13]. Bacteria production was estimated from algal production based on a model derived from data provided by Fouilland and Mostajir [17] while taking into account prediction error; *e.g.,* (BP; mg C m^−2^ d^−1^)  =  2.15*PPr^0^.^649^, n = 379, *r^2^* = 0.80, RMSE  =  0.348. The mid-line in the box and whisker plots represents the sample median, the filled box represents mean, the outer margins represent the 25^th^ and 75^th^ percentiles and the whiskers represent the 10^th^ and 90^th^ percentiles. Data sources provided in Table S1.

It is likely that the t-DOC flux values summarized in the analysis above were biased towards low values because limnologists tend to conduct field studies on lakes with HRTs between 1–5 yr, whereas many lakes, and especially lakes with high t-DOC fluxes, have much shorter HRTs. To account for this potential sampling bias, a distribution of lake areal hydrologic loadings rates, *i.e.,* lake inflow divided by lake surface area, obtained from a phosphorus mass balance analysis of 305 lakes [23] (*i.e.,* median  =  38 (11–153) L m^−2^ d^−1^) was multiplied by likely t-DOC concentrations based on stream surveys in Wisconsin, Ontario, Quebec, Nova Scotia, England, Scotland, Norway, Sweden and Finland (*i.e.,* t-DOC  =  11.1±7.4 mg L^−1^) (see Table S3). These calculations resulted in much larger t-DOC flux estimates than summarized in Fig. 1, *i.e.,* median  =  303 (80–1420) mg C m^−2^ d^−1^ vs. 62 (41–100) mg C m^−2^ d^−1^, respectively.

**Figure 2:**
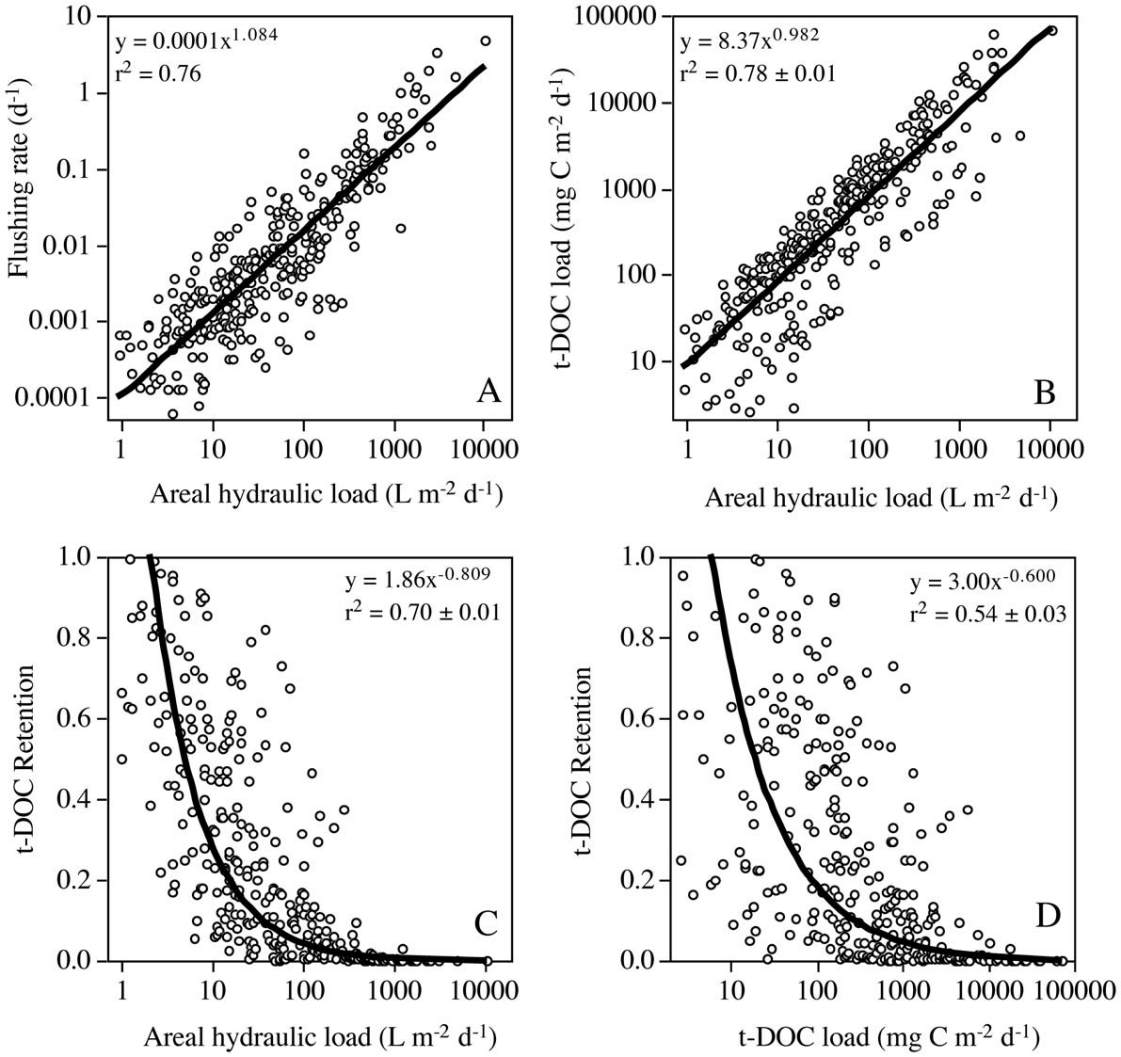
The statistical relationships among lake hydraulic loading, flushing, t-DOC loading and t-DOC retention. The lake morphometric and hydraulic characteristics used for these simulations were obtained for the lakes examined by Brett and Benjamin [23], see also Table S2. The input t-DOC concentration data were obtained from surveys of stream t-DOC concentrations conducted in northern North America and Europe, see also Table S2. Areal hydraulic loading is calculated as mean lake inflow divided by the lake surface area. This term is most commonly expressed as m yr^−1^, but is presented as L m^−2^ d^−1^ for simplicity. Lake flushing (ρ) is calculated as inflow divided by the lake volume. The areal t-DOC load is calculated as the hydraulic load multiplied by the input t-DOC concentration. Lake t-DOC retention is calculated as R  =  σ/(σ + ρ), where σ  =  0.0009±0.0004 d^−1^. A hybrid Bootstrap/Monte Carlo simulation approach was used to join the observed lake hydraulic data with a hypothetical distribution of input t-DOC concentrations (n = 305). These simulations were repeated 10 times to obtain confidence intervals for the coefficients of determination. Due to the dependency of the x and y ordinates in these plots, these statistical associations arise of mathematical necessity [47].

As previously noted, the flux of t-DOC to lakes is strongly correlated with areal hydraulic loading [20]–[22]. In any lake dataset with moderate variability, the areal hydraulic loading will also be strongly correlated with the lake’s HRT and its reciprocal, lake flushing (ρ) (Fig. 2A). The dependency between lake hydraulic loading and flushing, and the influence of hydraulic loading on the t-DOC influx (Fig. 2B), are important because flushing determines the residence time of t-DOC in lakes. The proportion of t-DOC removed within the lake, *e.g.,* due to photochemical oxidation, bacterial utilization or flocculation [30], is according to the continuously stirred tank reactor model calculated accordingly:
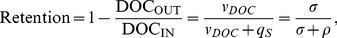
(1)where DOC_OUT_ is the lake water and outflow DOC concentration and DOC_IN_ is the flow weighted input concentration, *v*_DOC_ is an apparent t-DOC “settling velocity”, q_s_ is the areal hydraulic load, σ is the instantaneous first-order t-DOC loss rate, and ρ is the flushing rate [20]–[23].

To calculate the proportion of t-DOC retained within a particular lake, it is necessary to know the lake’s flushing rate, as well as the t-DOC degradation rate constant. Very few investigators have conducted complete t-DOC input budgets for specific lakes. However, Dillon and Molot [20]–[21] and Schindler et al. [22] conducted separate ≈20 yr t-DOC mass balance studies in multiple lakes making it possible to calculate the instantaneous t-DOC loss rate using very extensive long-term datasets, *i.e.,* σ  =  0.0009±0.0004 d^−1^ (± SD, n = 12). By comparison, in an eight month laboratory experiment Stets et al. [24] obtained data indicating a loss rate of 0.0013±0.0005 d^−1^ for the total DOC fraction in lake water (n = 12); and using an inverse modeling approach Algesten et al. [31] obtained results indicating σ  =  0.0018±0.0010 d^−1^ (n = 21). Summarizing these and other studies, Hanson et al. [32] concluded a wide range of evidence indicates that t-DOC in lakes degrades at ≈0.001 d^−1^. Because the t-DOC degradation rate derived from the long-term field studies [20]–[22] is based on complete input/output mass flux budgets that value is given precedence in our calculations.

One of the most important questions in aquatic science regards the role that lakes play in global carbon and greenhouse gas budgets [25], [31], [32]. If microbial or photochemical processes in lakes convert carbon inputs from the organic to the gas phase (*e.g.,* t-DOC to CO_2_ or CH_4_) lakes can be net sources of greenhouse gases. Headwater lakes may also be sources of CO_2_ if they receive substantial inputs of supersaturated groundwaters [33]. Algal exudates also contribute to the lake DOC pool, yet due to their highly labile biochemical composition this substrate is very preferentially and rapidly consumed by bacteria and does not persist [29]. This analysis, and particularly equation (1), shows the lake HRT primarily determines whether particular lakes act as sinks or conduits for terrestrial carbon inputs. According to the t-DOC degradation rate constant, in lakes with HRTs <3 yr the primary fate of t-DOC will be advection. Further, as is apparent from Fig. 2C, ≈78% of lakes used in this analysis have flushing rates that are larger than the average t-DOC loss rate. In a larger dataset of temperate lakes (n = 2025) where HRT was modeled a function of lake volume, watershed surface area and runoff, Webster et al. [34] (PA Soranno, unpubl. data) found 88% of lakes had HRTs <3 yr. Therefore, advection dominates the efflux of t-DOC from many lakes, and systems with high t-DOC loading rates primarily serve as conduits for downstream transport. Analyses of the role that lakes play in the global carbon and greenhouse gas cycling [25], [30] should account for the very strong influence of lake HRT on t-DOC advection [31], [32]. The present results indicate that t-DOC retention in lakes with HRTs of 0.1, 1 and 10 years average 3±1%, 22±10% and 63±28%, respectively. For the lake morphometric dataset considered in this study, t-DOC retention had a median value of 15% (3–45%), whereas the results of Algesten et al. [31] suggested a median of 51% (44–61%) for a suite of Swedish lakes. About 40% of this difference was due to the somewhat higher t-DOC degradation term associated with their data, but the difference was mostly due to the fact that the lake group they considered had much longer HRTs than the lake dataset used for this analysis, *i.e.,* median  =  2.1 (1.0–3.1 yr) vs 0.6 (0.1–2.6 yr), respectively. Hanson et al. [32] assumed a t-DOC loss rate similar to ours for many of their modeling scenarios (*i.e.,* σ  =  0.001 d^−1^), but their main lake dataset had HRTs values averaging 5.6±3.4 yr and they therefore concluded lakes process a much higher proportion of t-DOC than did the present study. Large compilations of lake morphometric properties show a clear majority (i.e., 60–65%) of temperate lakes have HRTs <1 yr [23], [34]. Thus the present analysis indicates t-DOC processing in lakes may be much less intense than commonly claimed, *i.e.,* R ≈50–70% [25], [30]–[32], because limnological field studies under-represent lakes with short HRTs.

Sobek et al. [35] analyzed data from 7,500 northern hemisphere lakes and concluded lake water DOC concentrations are regulated by a combination of catchment climatic, topographical, and lake properties. For example, lakes in catchments with low topographic relief and abundant wetlands tend to have higher DOC concentrations. The overall DOC concentrations for the dataset Sobek and colleagues [35] compiled had a median of 5.7 (2.7–10.4) mg L^−1^. As previously noted, the flushing rates of the lakes considered in this study indicated t-DOC removal has a median of 15% (3–45%), which suggests 50–70% of the between lake variation in lake DOC concentrations noted by Sobek and colleagues [35] could be due to differences in lake flushing rates. The importance of lake morphometric properties for t-DOC metabolism was foretold by Rasmussen et al. [36], who concluded that the color and t-DOC content of lake water tended to be higher in relatively small, shallow headwater lakes, with large and flat catchments, and short HRTs. del Giorgio and Peters [37] similarly emphasized the importance of lake HRT for t-DOC processing, when they noted lakes with large catchment to lake surface area ratios had high inputs of t-DOC, but low levels of in-lake t-DOC processing. These authors further hypothesized that the influence of HRT on lake t-DOC inputs and processing tended to cancel out and lakes had similar rates of t-DOC metabolism across a wide rage of conditions. Our analysis supports their hypothesis as absolute t-DOC loading was strongly (*r^2^* = 0.78) and moderately (*r^2^* = 0.55) positively correlated with areal hydraulic loading and flushing, respectively; whereas the flux of t-DOC removed in-lake had a very weak negative statistical association with the lake flushing rate (*r^2^* = 0.04).

### Total Versus Particulate Fluxes

The magnitude of allochthonous and autochthonous fluxes can be compared as the total or gross primary production that occurs within the lake relative to the total terrestrial carbon inputs that are processed within the lake. This approach has been used in the majority of studies on this topic [5], [8], [10], as well as the present study. Alternatively, one can compare only the particulate fluxes that stay suspended in the water column and are thus physically available for zooplankton consumption. If the latter approach is taken, it is necessary to distinguish between benthic and pelagic PPr. It is also necessary to account for the fact that the vast majority of the t-DOC that is removed in-lake is either photochemically oxidized, sedimented or metabolized very inefficiently by bacteria [29], [32], and thereby converted to CO_2_, and is thus unavailable to support zooplankton production. Similarly, t-POC fluxes to lakes are dominated by “leaves and buds” [10], which are several orders of magnitude larger than the particles zooplankton are able to ingest. Taking into account that 40–70% of the PPr in oligo/mesotrophic lakes is pelagic [38], bacteria convert about 10% of the t-DOC they process to biomass [29], and only 40% of t-POC inputs are small enough to be utilized by zooplankton [10], one ends up with an autochthonous basal resource flux that is many times larger than the corresponding allochthonous flux. Specifically, the median allochthonous particulate flux would be equal to ≈(10%)*34 mg m^−2^ d^−1^ = 3.4 mg m^−2^ d^−1^ for the bacteria production supported by t-DOC, and ≈(40%)*11 mg m^−2^ d^−1^ = 4.4 mg m^−2^ d^−1^ for ingestible sized t-POC inputs. Conversely, the median phytoplankton PPr would be ≈(40–70%)*253 mg m^−2^ d^−1^ = 101–177 mg m^−2^ d^−1^, giving an autochthonous particulate flux that is approximately 15–20 times larger than the allochthonous derived particulate flux.

We can compare the algal production rates we compiled to those from independent studies to assess whether these fluxes are reasonable. For example, the algal primary production values we summarized are somewhat less than the phytoplankton specific PPr rates that Wetzel [13] compiled for 25 oligo/mesotrophic lakes, *i.e.,* 285 (99–414) mg C m^−2^ d^−1^. Wetzel [13] further suggested typical phytoplankton PPr rates for oligo- and mesotrophic lakes range between 50–300 and 250–1000 mg C m^−2^ d^−1^, respectively. Vollenweider and Kerekes [39] used the OECD data set to develop a regression model that predicts areal phytoplankton production as a function of lake total phosphorus (TP; µg L^−1^) concentration accordingly:
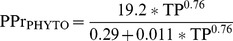
(2)


This equation predicts lakes with TP concentrations of 5, 10, 15 and 20 µg L^−1^, will have PPr of 199, 312, 399 and 471 mg C m^−2^ d^−1^, respectively. Because the results above are only for phytoplankton production, and the values we summarized were for benthic plus pelagic algal production, these independent approaches indicate the autochthonous production data we compiled are conservative. This conclusion is also supported by Lewis’ [40] recent analysis, which calculated the global areal production for undisturbed lakes averages ≈550 mg C m^−2^ d^−1^ when considering both pelagic and benthic production.

Another important factor to consider is whether the algal ^14^C uptake experiments, from which most of the lake PPr data we compiled were obtained, measure net or gross primary production. Several classic studies have concluded ^14^C uptake may underestimate gross PPr by a factor two particularly in oligotrophic systems [41], [42]. According to Wetzel [13] and Wetzel and Likens [43] most evidence “indicates that the ^14^C method measures photosynthetic rates closer to net photosynthesis than to gross”. Wetzel [13] and Lewis [40] also pointed out that the phytoplankton productivity released to the dissolved phase is approximately 20–25% of gross PPr. This suggests the total algal PPr values we compiled in our analysis were ≈15% too low, whereas the particulate flux generated from phytoplankton PPr was ≈10% too high.

**Figure 3:**
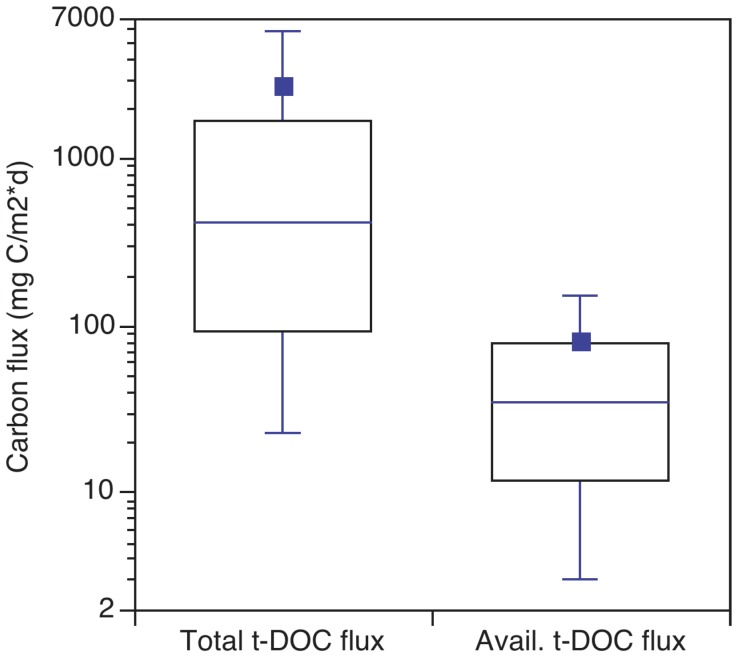
The influence of t-DOC loading and retention on absolute and available t-DOC fluxes. The absolute t-DOC loading values are from Fig. 2B. The available t-DOC flux was calculated as the absolute flux multiplied by the corresponding in-lake t-DOC retention from Fig. 2C, *i.e.,* (areal t-DOC loading)*(σ/(σ + ρ)).

Our conclusion that much of the t-DOC input to short HRT lakes is advected downstream is extremely important for the zooplankton allochthony hypothesis [1]–[5], because it is only the flux of t-DOC retained within lakes that may have been used to support food web processes. If the total t-DOC flux is corrected for retention, the mass flux of t-DOC that is removed in-lake is obtained (Fig. 3). This results in a factor ≈10 lower estimate of t-DOC availability, *i.e.,* 303 (80–1420) versus 34 (11–78) mg C m^−2^ d^−1^ (Fig. 3), because the lakes with the highest t-DOC loading rates also have very low removal (Fig. 2D). In fact, after accounting for removal, the available flux in the 32% of cases with absolute t-DOC loading greater than 1000 mg C m^−2^ d^−1^ declined from a median of 2661 (1552–7249) mg C m^−2^ d^−1^ to only 55 (23–137) mg C m^−2^ d^−1^, indicating 98% of t-DOC is advected from the lakes with the highest areal t-DOC loading rates. Terrestrially derived DOC that is removed in-lake may be photochemically degraded, flocculated and subsequently sedimented, or metabolized by bacteria to produce greenhouse gases (CO_2_ or CH_4_), or new cells [30]. However, the growth efficiency of bacteria utilizing t-DOC is very low [29] and both t-DOC derived flocs and bacteria are likely very low nutritional quality resources for zooplankton [6], [18], [19]. These mass flux calculations also indicate the total flux of available terrestrial inputs will be approximately a factor 4–7 smaller than rates of algal primary production in typical oligo/mesotrophic lakes; *i.e.,* 48 (26–89) mg C m^−2^ d^−1^ vs 253 (115–546) mg C m^−2^ d^−1^, respectively. Conversely, del Giorgio and Peters [37] challenged the traditional phytoplankton photosynthesis paradigm in limnology and concluded that in the oligotrophic and mesotrophic systems they sampled, phytoplankton production was often only a minor fraction of whole lake carbon metabolism. These different conclusions were mainly because del Giorgio and Peters [37] assumed all of their lakes had input t-DOC concentrations of 24 mg C L^−1^ based on watershed carbon yields [44], whereas our meta-analysis of temperate and boreal streams indicated concentrations of 11.1±7.4 mg C L^−1^. del Giorgio and Peters’ high assumed input t-DOC concentration increased their reported available t-DOC flux, and the t-DOC degradation rate derived from their data, by a factor of 3.5±0.7. If the results of their study are recalculated with 11.1 mg L^−1^ as the input t-DOC concentration, t-DOC metabolism as a percent of phytoplankton production for their lakes declines from 139% (95–275%) to 37% (29–93%).

### Allochthonous Support of Zooplankton Production

**Figure 4:**
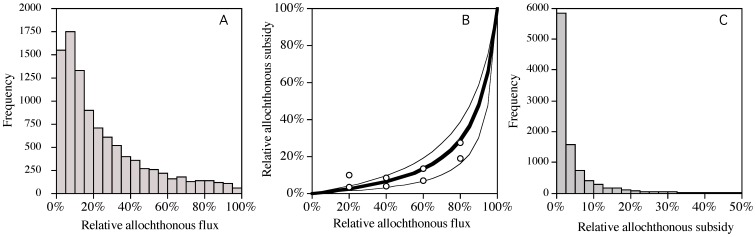
A comparison of the relative magnitude of available allochthonous and autochthonous resources, the relative food quality of these resources, and the predicted allochthonous subsidy to zooplankton production after accounting for resource quantity and quality. Panel A, the distribution of the percent of basal resources from allochthonous sources is depicted in the histogram. Panel B, the functional response showing the percent of aquatic herbivore production that is expected to be supported by terrestrial sources at a particular relative available allochthonous flux. This functional response was derived from the fatty acid profiles of *Daphnia* fed mixed diets comprised of allochthonous and autochthonous resources as reported in Brett et al. [6]. The dark line is based on *Daphnia* utilizing carbon 10.2% as efficiently as phytoplankton and the thin lines represent ±5.5% (SD) uncertainty. The white points represent the estimated terrestrial contributions to zooplankton from the gradient experiment [6]. Panel C, the distribution of expected zooplankton allochthony values based on the availability of allochthonous resources depicted in panel A and the food quality/preference functional response depicted in panel B.

As noted earlier, due to the very low food quality of terrestrial resources the flux of this basal resource would need to be considerably larger than algal production in order to make a substantial contribution to zooplankton production [5], [6]. Our calculations show that after accounting for t-DOC advection, the flux of available t-DOC and t-POC is a small portion of the total available resources compared to algal production, *i.e.,* 18% (9–34%) (Fig. 4A). Further, much of the t-DOC that is removed within lakes will be mineralized directly by photolysis, respired to CO_2_ by bacteria or lost to the sediment [30]–[32] without contributing to the eukaryotic portion of the food web.

A recent study showed that *Daphnia* fed diets comprised entirely of terrestrially derived matter had lower growth efficiencies (5 vs. 41%), reproduced later (19.4 vs. 13.5 d), had fewer neonates (3.1 vs. 69.5) and were smaller (0.22 vs. 1.06 mg dry wt. ind.^−1^) than those that consumed phytoplankton [6]. However, in natural systems freshwater zooplankton will generally consume mixtures of autochthonous and allochthonous resources. Thus, the physiological responses of zooplankton to mixed diets are the most relevant to the current analysis. Brett et al. [6] carried out a gradient experiment, where *Daphnia* were fed t-POC and phytoplankton in 20% increments (Table S4). The fatty acid profiles of *Daphnia* that consumed pure diets were used to calculate preferential utilization of phytoplankton when *Daphnia* consumed mixed diets. The fatty acid profiles of *Daphnia* consuming pure t-POC and phytoplankton were very different (*i.e., r^2^* = 0.09), but the preferential utilization calculations provided very strong fits to the observed fatty acid profiles of the zooplankton consuming mixed diets (*r^2^*≈0.99). These solutions also indicated very preferential phytoplankton utilization. Two end-member mixing models based on *Daphnia* fatty acid ω3:ω6 ratios can also be used to infer selective resource utilization. For example, *Daphnia* that consumed 100% t-POC had a ω3:ω6 ratio of 1.6, *Daphnia* that consumed 100% *Cryptomonas* had a ratio of 11.7, and *Daphnia* that consumed mixed diets had ω3:ω6 ratios of 8.9–10.8. Altogether, these data indicate *Daphnia* preferentially utilized phytoplankton by a factor of 11.8±5.8 or alternatively utilized terrestrial resources 10±5% as efficiently. According to these outcomes, in order for zooplankton to obtain 30–70% of their resources from terrestrial inputs as many studies have concluded [1]–[5], terrestrial influxes would have to be 89±8% of total resources. Brett et al. [6] also showed that when *Daphnia* consumed mixed diets of t-POC and phytoplankton, they had substantially higher growth efficiencies on the allochthonous portion of their diet (*i.e.,*  = 20%) than when t-POC was the sole resource (*i.e.,* 5%) [6]. Because *Daphnia* consuming pure phytoplankton diets had ≈40% growth efficiency, the results above suggest *Daphnia* consuming 50∶50 t-POC and phytoplankton should have obtained 32% of their resources from the t-POC. However, the fatty acid profiles of the *Daphnia* in this treatment indicate they only obtained 12% of their lipids for the terrestrial resource (unpublished data). As noted in Brett et al. [6], this may suggest zooplankton are able to realize a catabolic benefit (*i.e.,* obtain energy) when they utilize low quality terrestrial resources even if this matter is not used for anabolic processes (*i.e.,* building new biomass).

We used the available basal resource fluxes (Fig. 4A) and the zooplankton functional response to terrestrial resources (Fig. 4B) to calculate an expected zooplankton allochthony for oligo/mesotrophic lakes. When considering both the low quantity and quality of allochthonous resources, our calculations indicate aquatic herbivores are likely to obtain 1.8% (0.6–5.2%) of their resources from terrestrial inputs (Fig. 4C). If these calculations are based solely on the particulate fluxes that would be available to grazing zooplankton, the calculated allochthonous support of zooplankton production would be smaller because most of the t-POC loaded to lakes is too large for zooplankton to ingest and bacteria utilize t-DOC very inefficiently.

### Conclusions

Although obtained for a particular set of conditions, the results of this analysis should be robust provided: 1) lake hydraulic loading and flushing are highly correlated, 2) areal t-DOC loading is strongly dependent on hydraulic loading, 3) t-DOC degrades at ≈0.1% d^−1^, and 4) terrestrially derived particulate matter is a very low quality resource for zooplankton and other herbivorous metazoans. These conditions are likely to be true for many north temperate lakes. It should be noted that t-DOC inputs affect the physical (*e.g.,* light and temperature zonation) and chemical (*e.g.,* dissolved oxygen concentrations and nutrient bioavailability) properties of lakes in ways that are very important for the overall functioning of the system [35], [45]. In particular, high in-lake t-DOC concentrations may strongly inhibit autochthonous primary production by inducing severe light limitation [46]. However, the present results indicate terrestrial inputs are likely to make very small direct contributions to animal production in most lakes.

## Methods

### Basal Resource Mass Fluxes

Several high profile narrative reviews have concluded there is overwhelming evidence terrestrial inputs dominate the carbon budgets of many oligotrophic lakes [2], [5], [12]–[15]. However, no study has comprehensively analyzed the lake carbon flux literature to quantitatively test this conclusion. We conducted a quantitative analysis of the lake literature to statistically assess the empirical basis for this generality. The data from field studies conducted at the UNDERC lakes [8], [10], [26], [27] were also compared against the model assumptions used to represent the carbon budgets of the same lake group [3], [8]. Our quantitative analysis of terrestrial carbon fluxes to lakes, in relation to autotrophic primary production within lakes, was carried out by summarizing t-DOC loading rates for all field studies which have directly determined t-DOC input concentrations and hydraulic loading rates (Table S1 and References S1). We did not use the results of analyses that indirectly estimated t-DOC loading based on catchment land-cover and vegetation type; *i.e.,* studies that did not use actual field measurements of input t-DOC concentrations [8], [9], [31], [32], [37]. Autochthonous primary production was quantified for all studies that we are aware of that directly determined the production rate of both phytoplankton and benthic algae in lakes with total phosphorus concentrations ≤20 µg L^−1^ (Table S2 and References S1). Bacterial production was estimated from algal production according to a model derived from data provided by Fouilland and Mostajir [17]; *e.g.,*

(BP; mg C m^−2^ d^−1^)  =  2.15*PPr^0⋅649^, n = 379, *r^2^* = 0.80, RMSE  =  0.348.

### Hydraulic Flushing and t-DOC Loading and Retention

Terrestrial DOC loading is the product of the input t-DOC concentration multiplied by the areal hydraulic loading rate (*i.e.,* q_s_  =  lake inflows/lake surface area). Lake input t-DOC concentrations varying arithmetically, whereas lake hydraulic loading rates vary logarithmically. Therefore, t-DOC loading should be strongly correlated with q_s_ and lakes with very short HRTs will generally have much higher t-DOC loading. During our analysis of field studies that directly determined t-DOC loading to lakes, it was apparent that this database was comprised primarily of lakes with HRTs ranging between 1–5 yr. Conversely, in a large dataset of lakes (n = 305) used to assess phosphorus input/output budgets [23], 25% of lakes had HRTs <0.1 year and 58% had HRTs <1 year. The lake HRT distribution for this dataset had the same median (i.e., 0.58 yr), but somewhat wider tails in geometric space than the larger population of lakes (n = 2025) considered by Webster et al. [34] (PA Soranno, unpubl. data). To test whether typical lakes have on average larger t-DOC loading than the lakes usually sampled in limnological field studies, we used the distribution of lake q_s_ values obtained from Brett and Benjamin [23], as well as directly determined stream t-DOC concentrations for a large number of systems in North America and northern Europe (Table S3 and References S1), to generate a hypothetical distribution of lake t-DOC loading rates that accounts for the high prevalence of short HRT lakes. The q_s_ values in this distribution could be approximately represented by a cumulative probability density function based on a sigmoid type response; *i.e.,* q_s_ (L m^−2^ d^−1^)  =  a*x/(1−*x*), where *a* is a coefficient with a value of 45.8 L m^−2^ d^−1^, and *x* is the percentile between 0–1, *r^2^* = 0.98, n = 305.

According to the classic mass balance equations for lakes [23], constituents can either be removed within or advected from a lake. In the case of t-DOC, removal can be due to flocculation and accumulation in the sediments, photochemical oxidation or bacterial metabolism [30]–[32]. However, according to the Principle of Mass Conservation, a constituent cannot be both removed and advected from a system; therefore, that t-DOC loading to lakes which is ultimately advected cannot also be used to support in-lake food web processes. Because lake t-DOC inputs are strongly associated with hydraulic flushing, lakes with short HRTs are likely to have very high t-DOC inputs as well as similarly high t-DOC advective losses. We characterized the relationship between lake hydraulic flushing, t-DOC loading, in-lake t-DOC removal and advection using the lake morphometric properties and input t-DOC concentrations previously mentioned. In-lake t-DOC removal was quantified using the classic mass balance equation [23] accordingly: Removal  =  σ/(σ + ρ), where σ represents the first order loss rate for t-DOC and ρ represents advective losses from the lake.

### Calculating Potential Zooplankton Allochthony

Recent research has shown that terrestrial carbon inputs to lakes are, due to their biochemical composition and recalcitrance, very low food quality resources for herbivorous zooplankton [6]. Therefore, a fixed amount of terrestrially derived resources will support far less aquatic herbivore production than would an equivalent amount of algae [5]. To account for this food quality effect, we used the available basal resource fluxes (*i.e.,* t-POC inputs + available t-DOC inputs) and the autochthonous production depicted in Fig. 1, as well as the respective quality of these fluxes, to calculate the proportion of zooplankton production that would be expected to be supported by typical allochthonous and autochthonous basal resource fluxes. Specifically, the expected proportion of aquatic herbivore production supported by allochthonous basal resource influxes was calculated accordingly:
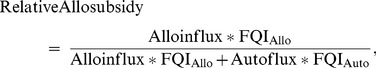
(3)where Allo influx equals the areal influx of available basal resources, FQI_Allo_ equals a food quality index for allochthonous resources, Auto flux equals autochthonous PPr, and FQI_Auto_ equals the food quality index for autochthonous resources.

We used the outcomes of an experiment where *Daphnia* were fed a gradient of allochthonous and autochthonous resources to calculate preferential utilization of phytoplankton when these zooplankters utilized mixed diets (Table S4). As explained in Brett et al. [6], the fatty acid profiles of *Daphnia* that consumed 100% t-POC and phytoplankton diets where used to reverse-engineer the contributions of allochthonous resources to the *Daphnia* fed mixed diets based on the fatty acid composition of these daphnids. For example, *Daphnia* that were fed 80% t-POC and 20% phytoplankton obtained 81% of their fatty acids from phytoplankton. For the present study, we also used the *Daphnia* fatty acid ω3:ω6 ratios and two end-member mixing models to calculate selective resource utilization. This was based on the fact that *Daphnia* that consumed 100% t-POC had a low ω3:ω6 ratio (*i.e.,* 1.6), whereas *Daphnia* that consumed 100% *Cryptomonas* had a very high ratio (*i.e.,* 11.7) and *Daphnia* that consumed mixed diets had intermediate ratios (*i.e.,* 8.9–10.8). Once values for allochthonous contributions to the zooplankton were obtained, these were fit to equation (3) to derive FQI_Allo_ values, assuming a baseline of FQI_Auto_  = 1. Different FQI_Allo_ values were fit based on the total fatty acid profile and ω3:ω6 ratio results. Optimal fits were obtained by minimizing the error sum of squares between observed and predicted values using the Solver function in Microsoft Excel. Because our resource flux calculations showed allochthonous resources were <50% of the total available resources in 84% of cases, we also fit these results to obtain FQI_Allo_ values using only the cases where t-POC was <50% of the available food in the gradient experiment [6]. These calculations were carried out within a Monte Carlo simulation framework by randomly pairing individual t-POC and available t-DOC influx estimates with autochthonous production values (n = 10,000). We also used a Monte Carlo approach to combine the calculated allochthonous and phytoplankton fluxes with the fitted distribution of FQI_Allo_ values using equation (3) to calculate the expected zooplankton allochthony when considering both food quantity and quality constraints (n = 10,000). This approach assumes all t-DOC removed within lakes is “available” to support upper trophic level processes, whereas most of the t-DOC metabolized by bacteria is transformed to CO_2_ via respiration [29]. Because this calculation accounts for both pelagic and benthic primary production, it represents potential allochthonous subsidies to both benthic and pelagic herbivores and detritivores and not zooplankton exclusively. Calculations that only consider the particulate fluxes available to zooplankton would indicate less zooplankton allochthony.

## Supporting Information

Table S1 **Allochthonous influxes.**
(DOC)

Table S2 **Autochthonous production.**
(DOC)

Table S3 **Stream t-DOC concentrations.**
(DOC)

Table S4 **Daphnia fatty acid composition.**
(DOC)

References S1 (DOC)

## References

[1] Grey J, Jones RI, Sleep D (2001). Seasonal changes in the importance of the source of organic matter to the diet of zooplankton in Loch Ness, as indicated by stable isotope analysis.. Limnol Oceanogr.

[2] Karlsson J, Jonsson A, Meili M, Jansson M (2003). Control of zooplankton dependence on allochthonous organic carbon in humic and clear-water lakes in northern Sweden.. Limnol Oceanogr.

[3] Pace ML, Cole JJ, Carpenter SR, Kitchell JF, Hodgson JR (2004). Whole-lake carbon-13 additions reveal terrestrial support of aquatic food webs.. Nature.

[4] Jansson M, Persson L, De Roos AM, Jones RI, Tranvik LJ (2007). Terrestrial carbon and intraspecific size-variation shape lake ecosystems.. Trends Ecol Evol.

[5] Cole JJ, Carpenter SR, Kitchell J, Pace ML, Solomon CT (2011). Strong evidence for terrestrial support of zooplankton in small lakes based on stable isotopes of carbon, nitrogen, and hydrogen.. Proc Natl Acad Sci USA.

[6] Brett MT, Kainz MJ, Taipale SJ, Seshan H (2009). Phytoplankton, not allochthonous carbon, sustains herbivorous zooplankton production.. Proc Natl Acad Sci USA.

[7] Francis TB, Schindler DE, Holtgrieve GW, Larson ER, Scheuerell MD (2011). Habitat structure determines resource use by zooplankton in temperate lakes.. Ecol Lett.

[8] Cole JJ, Carpenter SR, Pace ML, Van de Bogert MC, Kitchell JL (2006). Differential support of lake food webs by three types of terrestrial organic carbon.. Ecol Lett.

[9] Berggren M, Strom L, Laudon H, Karlsson J, Jonsson A (2010). Lake secondary production fueled by rapid transfer of low molecular weight organic carbon from terrestrial sources to aquatic consumers.. Ecol Lett 13, 870–880.

[10] Preston ND, Carpenter SR, Cole JJ, Pace ML (2008). Airborne carbon deposition on a remote forested lake.. Aquat Sci.

[11] Pace ML, Carpenter SR, Cole JJ, Coloso JJ, Kitchell JF (2007). Does terrestrial organic carbon subsidize the planktonic food web in a Clearwater lake?. Limnol Oceanogr.

[12] Wetzel RG (1995). Death, detritus and energy flow in aquatic ecosystems.. Freshwat Biol.

[13] Wetzel RG (2001). Academic Press..

[14] Prairie YT (2008). Carbocentric limnology: looking back, looking forward.. Can J Fish Aquat Sci.

[15] Reynolds CS (2008). A changing paradigm of pelagic food webs.. Int Rev Hydrobio.

[16] Fenchel T (2008). The microbial loop - 25 years later.. J Exp Mar Bio Ecol.

[17] Fouilland E, Mostajir B (2010). Revisited phytoplanktonic carbon dependency of heterotrophic bacteria in freshwaters, transitional, coastal and oceanic waters.. FEMS Microbiol Ecol.

[18] Martin-Creuzburg D, Beck B, Freese HM (2011). Food quality of heterotrophic bacteria for *Daphnia magna*: evidence for a limitation by sterols.. FEMS Microbiol Ecol.

[19] Taipale SJ, Kainz MJ, Brett MT (2012). The influence of bacteria dominated diets on *Daphnia magna* somatic growth, reproduction, and lipid composition.. FEMS Microbiol Ecol DOI.

[20] Dillon PJ, Molot LA (1997). Dissolved organic and inorganic carbon mass balances in central Ontario lakes.. Biogeochemistry.

[21] Dillon PJ Molot LA 2005 Long-term trends in catchment export and lake retention of dissolved organic carbon, dissolved organic nitrogen, total iron, and total phosphorus: The Dorset, Ontario, study, 1978–1998. J Geophys Res 110, G01002, doi 10.1029/2004JG000003

[22] Schindler DW, Curtis PJ, Bayley SE, Parker BR, Beaty KG (1997). Climate-induced changes in the dissolved organic carbon budgets of boreal lakes.. Biogeochemistry.

[23] Brett MT, Benjamin MM (2008). A reassessment of lake phosphorus retention and the nutrient loading concept in limnology.. Freshw Biol.

[24] Stets EG, Striegl RG, Aiken GR (2010). Dissolved organic carbon export and internal cycling in small, headwater lakes.. Global Biogeochem Cy.

[25] Cole JJ, Prairie YT, Caraco NF, McDowell WH, Tranvik LJ (2007). Plumbing the global carbon cycle: Integrating inland waters into the terrestrial carbon budget.. Ecosystems.

[26] Christensen DL, Carpenter SR, Cottingham KL, Knight SE, LeBouton JP (1996). Pelagic responses to changes in dissolved organic carbon following division of a seepage lake.. Limnol Oceanogr.

[27] Cole JJ, Pace ML (1998). Hydrologic variability of small, northern Michigan lakes measured by the addition of tracers.. Ecosystems.

[28] Vano JA Foley JA Kucharik CJ Coe MT 2006 Evaluating the seasonal and interannual variations in water balance in northern Wisconsin using a land surface model. J Geophys Res Biogeosci 111: G02025, doi 10.1029/2005JG000112

[29] del Giorgio PA, Cole JJ (1998). Bacterial growth efficiency in natural aquatic systems.. Ann Rev Ecol Syst.

[30] Tranvik JJ, Downing JA, Cotner JB, Loiselle SA, Striegl RG (2009). Lakes and reservoirs as regulators of carbon cycling and climate.. Limnol Oceanogr.

[31] Algesten G, Sobek S, Bergstrom AK, Ågren A, Tranvik LJ (2004). Role of lakes for organic carbon cycling in the boreal zone.. Global Change Biol.

[32] Hanson PC, Hamilton DP, Stanley EH, Preston N, Langman OC (2011). Fate of allochthonous dissolved organic carbon in lakes: A quantitative approach. PLoS ONE 6: e21884..

[33] Dubois K, Carignan R, Veizer J (2009). Can pelagic net heterotrophy account for carbon fluxes from eastern Canadian lakes?. Appl Geochem.

[34] Webster KE, Soranno PA, Cheruvelil KS, Bremigan MT, Downing JA (2008). An empirical evaluation of the nutrient-color paradigm for lakes.. Limnol Oceanogr.

[35] Sobek S, Tranvik LJ, Prairie YT, Kortelainen P, Cole JJ (2007). Patterns and regulation of dissolved organic carbon: An analysis of 7,500 widely distributed lakes.. Limnol Oceanogr.

[36] Rasmussen JB, Godbout L, Schallenberg M (1989). The humic content of lake water and its relationship to watershed and lake morphometry.. Limnol Oceangr.

[37] del Giorgio PA, Peters RH (1994). Patterns in planktonic P:R ratios in lakes: influence of lake trophy and dissolved organic carbon.. Limnol Oceanogr.

[38] Vadeboncoeur Y, Steinman AD (2002). Periphyton function in lake ecosystems.. TheScientificWorldJOURNAL.

[39] Vollenweider RA, Kerekes J (1980). Loading concept as basis for controlling eutrophication: Philosophy and preliminary results of the OECD Programme on Eutrophication.. Prog Water Technol.

[40] Lewis WM (2011). Global primary production of lakes: 19^th^ Baldi Memorial Lecture.. Inland Waters.

[41] Peterson BJ (1980). Aquatic primary productivity and the ^14^C-CO_2_ method: a history of the productivity problem.. Ann Rev Ecol Syst.

[42] Carignan R, Planas D, Vis C (2000). Planktonic production and respiration in oligotrophic shield lakes.. Limnol Oceangr.

[43] Wetzel RG, Likens GE (2000). Limnological Analyses..

[44] Eckhardt BW, Moore TR (1990). Controls on dissolved organic carbon concentrations in streams, southern Québec.. Can J Fish Aquat Sci.

[45] Steinberg CEW, Kamara S, Prokhotskaya VY, Manusadzianas L, Karasyova TA (2006). Dissolved humic substances - ecological driving forces from the individual to the ecosystem level?. Freshw Biol.

[46] Jones RI (1992). The influence of humic substances on lacustrine planktonic food chains.. Hydrobiologia.

[47] Brett MT (2004). When is a correlation between ratios “spurious”?. Oikos.

