# Supplementary material for: Mass Flux Calculations Show Strong Allochthonous Support of Freshwater Zooplankton Production Is Unlikely
Source: PLoS One. 2012 Jun 26;7(6):e39508. doi: 10.1371/journal.pone.0039508 (PMC3383696; doi:10.1371/journal.pone.0039508)
Supplement: References S1 — (DOC) [file pone.0039508.s005.doc]

**References S1**

1. Dillon PJ, Molot LA (1997) Dissolved organic and inorganic carbon mass balances in central Ontario lakes. Biogeochemistry 36: 29-42.

2. Dillon PJ, Molot LA (2005) Long-term trends in catchment export and lake retention of dissolved organic carbon, dissolved organic nitrogen, total iron, and total phosphorus: The Dorset, Ontario, study, 1978–1998. J Geophys Res 110, G01002, doi:10.1029/2004JG000003.

3. Schindler DW, Curtis PJ, Bayley SE, Parker BR, Beaty KG, et al. (1997) Climate-induced changes in the dissolved organic carbon budgets of boreal lakes. Biogeochemistry 36: 9-28.

4. Christensen DL, Carpenter SR, Cottingham KL, Knight SE, LeBouton JP, et al. (1996) Pelagic responses to changes in dissolved organic carbon following division of a seepage lake. Limnol Oceanogr 41: 553-559.

5. Cole JJ, Pace ML (1998) Hydrologic variability of small, northern Michigan lakes measured by the addition of tracers. Ecosystems 1: 310-320.

6. Wetzel RG (2001) Chapter 23, Detritus: organic carbon cycling and ecosystem metabolism. In Limnology, Lake and River Ecosystems, 3^rd^ edition. Academic Press.

7. Whalen SC, Cornwell JC (1985) Nitrogen, phosphorus, and organic carbon cycling in an arctic lake. J Can Fish Aquat Sci 42: 797-808.

8. Sobek S, Söderbäck B, Karlsson S, Andersson E, and Brunberg AK (2006) A carbon budget of a small humic lake: an example of the importance of lakes for organic matter cycling in boreal catchments. Ambio 35:469-475.

9. Stets EG, Striegl RG, Aiken GR (2010). Dissolved organic carbon export and internal cycling in small, headwater lakes. Global Biogeochem Cy 24: 1-12.

10. Preston ND, Carpenter SR, Cole JJ, Pace ML (2008) Airborne carbon deposition on a remote forested lake. Aquat Sci 70: 213-224.

11. Vadeboncoeur Y, Steinman AD (2002) Periphyton function in lake ecosystems. TheScientificWorldJOURNAL 2:1449-1468.

12. Vadeboncoeur Y, Jeppesen M, Jake Vander Zanden MJ, Schierup H-H, Christoffersen K, Lodge DM (2003) From Greenland to green lakes: Cultural eutrophication and the loss of benthic energy pathways in lakes. Limnol Oceanogr 48:1408-1418.

13. Vander Zanden MJ, Chandra S, Park S, Vadeboncoeur Y, Goldman CR (2006) Efficiencies of benthic and pelagic trophic pathways in a subalpine lake. Can Fish Aquat Sci 63: 2608-2620.

14. Cole JJ, Carpenter SR, Pace ML, Van de Bogert MC, Kitchell JL, et al. (2006) Differential support of lake food webs by three types of terrestrial organic carbon. Ecol Lett 9: 558-568.

15. Whalen SC, Chalfant BA, Fischer EN (2008) Epipelic and pelagic primary production in Alaskan Arctic lakes of varying depth. Hydrobiologia 614: 243–257.

16. Karlsson J, Byström P, Ask J, Ask P, Persson L, Jansson M (2009) Light limitation of nutrient-poor lake ecosystems. Nature 460: 506-509.

17. Eckhardt BW, Moore TR (1990) Controls on dissolved organic-carbon concentrations in streams, southern Quebec. Can J Fish Aquat Sci 47: 1537-1544.

18. Molot LA, Dillon PJ (1997) Colour-mass balances and colour-dissolved organic carbon relationships in lakes and streams in central Ontario. Can J Fish Aquat Sci 54: 2789-2795.

19. Mulholland PJ (1997) Dissolved organic matter concentration and flux in streams. J North Am Benthol Soc 16: 131-141.

20. Gorham E, Underwood JK, Janssens JA, Freedman B, Maass W, et al. (1998) The chemistry of streams in southwestern and central Nova Scotia, with particular reference to catchment vegetation and the influence of dissolved organic carbon primarily from wetlands. Wetlands 18: 115-132.

21. Gergel SE, Turner MG, Kratz TK. (1999) Dissolved organic carbon as an indicator of the scale of watershed influence on lakes and rivers. Ecol Appl 9: 1377-1390.

22. Kortelainen P, Mattsson T, Finer L, Ahtiainen M, Saukkonen S, et al. (2006) Controls on the export of C, N, P and Fe from undisturbed boreal catchments, Finland. Aquat Sci 68: 453-468.

23. Aitkenhead-Peterson JA, Smart RP, Aitkenhead MJ, Cresser MS, McDowell WH (2007) Spatial and temporal variation of dissolved organic carbon export from gauged and ungauged watersheds of Dee Valley, Scotland: Effect of land cover and C:N. Water Resour Res 43, W05442, doi:10.1029/2006WR004999.

24. Baker A, Cumberland S, and Hudson N (2008) Dissolved and total organic and inorganic carbon in some British rivers. Area 40: 117–127.

25. Strand LT, Haaland S, Kaste O, Stuanes AO (2008) Natural variability in soil and runoff from small headwater catchments at Storgama, Norway. Ambio 37: 18-28.

26. Löfgren S, Cory N, Zetterberg T (2010) Aluminium concentrations in Swedish forest streams and co-variations with catchment characteristics. Environ Monit Assess 166: 609-624.

27. Brett MT, Kainz MJ, Taipale SJ, Seshan H (2009) Phytoplankton, not allochthonous carbon, sustains herbivorous zooplankton production. Proc Natl Acad Sci USA 106: 21197-21201.
