# Supplementary material for: Mass Flux Calculations Show Strong Allochthonous Support of Freshwater Zooplankton Production Is Unlikely
Source: PLoS One. 2012 Jun 26;7(6):e39508. doi: 10.1371/journal.pone.0039508 (PMC3383696; doi:10.1371/journal.pone.0039508)
Supplement: Supplementary file 2 [file pone.0039508.s001.doc]

Table S1. Lake terrestrial resource influx estimates (mg C m^{-2} d^{-1}).

Flux	Rate	source
t-DOC		
	18	1,2
	97	1,2
	109	1,2
	85	1,2
	79	1,2
	41	1,2
	64	1,2
	80	3
	54	3
	115	3
	54	3
	39	3
	38	3
	60	4,5
	57	6
	29	6
	265	7
	203	8
	7.1	9
	211	9
Aeolian t-POC (large and small size fractions)		
	44	10
	12	10
	4.4	10
	5.3	10
	10	10
	8.2	10
	1.9	10
	2.4	10
Fluvial t-POC		
	10% of t-DOC flux	6
