# Supplementary material for: Mass Flux Calculations Show Strong Allochthonous Support of Freshwater Zooplankton Production Is Unlikely
Source: PLoS One. 2012 Jun 26;7(6):e39508. doi: 10.1371/journal.pone.0039508 (PMC3383696; doi:10.1371/journal.pone.0039508)
Supplement: Supplementary file 3 [file pone.0039508.s002.doc]

Table S2. Lake pelagic and benthic primary production estimates (mg C m$^{-2}$ d$^{-1}$).

| Flux | Rate | source |
|---|---|---|
| PPr | | |
| | 60 | 11 |
| | 30 | 11 |
| | 256 | 11 |
| | 1072 | 11 |
| | 257 | 11 |
| | 162 | 11 |
| | 205 | 11 |
| | 546 | 11 |
| | 335 | 11 |
| | 545 | 11 |
| | 70 | 11 |
| | 635 | 11 |
| | 183 | 11 |
| | 1014 | 12 |
| | 305 | 12 |
| | 158 | 12 |
| | 129 | 12 |
| | 519 | 12 |
| | 144 | 12 |
| | 266 | 12 |
| | 398 | 12 |
| | 686 | 12 |
| | 696 | 12 |
| | 767 | 12 |
| | 556 | 12 |
| | 614 | 12 |
| | 212 | 12 |
| | 920 | 12 |
| | 1216 | 12 |
| | 631 | 12 |
| | 631 | 12 |
| | 1043 | 12 |
| | 201 | 12 |
| | 464 | 12 |
| | 255 | 12 |
| | 444 | 13 |
| | 551 | 14 |
| | 446 | 14 |
| | 409 | 14 |
| | 485 | 10 |
| | 120 | 15 |
| | 252 | 15 |
| | 113 | 15 |
| | 107 | 15 |
| | 82 | 15 |
| | 79 | 15 |
| | 4.5 | 16 |
| | 10 | 16 |
| | 33 | 16 |
| | 65 | 16 |
| | 93 | 16 |
| | 99 | 16 |
| | 109 | 16 |
| | 114 | 16 |
| | 120 | 16 |
| | 132 | 16 |
| | 194 | 16 |
| | 200 | 16 |
