# Supplementary material for: Mass Flux Calculations Show Strong Allochthonous Support of Freshwater Zooplankton Production Is Unlikely
Source: PLoS One. 2012 Jun 26;7(6):e39508. doi: 10.1371/journal.pone.0039508 (PMC3383696; doi:10.1371/journal.pone.0039508)
Supplement: Supplementary file 4 [file pone.0039508.s003.doc]

Table S3. Stream DOC concentrations (mg L^{-1} ± 1 SD).

	Mean	± SD	Source
Quebec	15.9	14.1	17
Ontario	8.9	4.2	18
North America	9.5	10.3	19
Nova Scotia	17.5	9.5	20
Wisconsin	10.0	6.9	21
Finland	13.1	8.3	22
Scotland	4.5	2.3	23
England	8.7	6.0	24
Norway	9.1	4.1	25
Sweden	14.0	2.1	26
Overall mean	11.1	7.4	
