# Supplementary material for: Mass Flux Calculations Show Strong Allochthonous Support of Freshwater Zooplankton Production Is Unlikely
Source: PLoS One. 2012 Jun 26;7(6):e39508. doi: 10.1371/journal.pone.0039508 (PMC3383696; doi:10.1371/journal.pone.0039508)
Supplement: Supplementary file 5 [file pone.0039508.s004.doc]

Table S4. The FA composition of *Daphnia* fed a gradient of allochthonous and autochthonous resources. See Brett et al. [27] for a detailed description of methods.

Sample	Diet	Daph	Daph	Daph	Daph	Daph	Daph	Diet
Fatty acid	t-POC	100%	80%	60%	40%	20%	0%	
		0%	20%	40%	60%	80%	100%	Phyto
C14:0	4.3	2.2	1.5	1.2	1.1	1.0	1.0	1.0
C15:0	0.1	0.7	0.6	0.5	0.4	0.4	0.3	0.0
C16:0	49.9	18.6	15.5	13.0	12.2	11.8	11.2	13.8
C16:1 ω7	1.9	19.0	3.5	1.4	1.0	0.8	0.7	0.8
X17:0	0.4	0.9	2.2	2.0	2.0	2.0	1.9	0.4
X18:0	8.3	2.9	3.6	3.3	3.3	3.1	3.3	0.8
X18:1 ω9	2.9	13.9	5.5	4.7	4.5	5.2	4.2	1.2
X18:2 ω6	5.1	13.5	4.6	3.9	3.6	3.6	3.4	3.4
X18:3 ω6	0.0	0.0	0.2	0.5	0.5	0.6	0.6	0.0
X18:3 ω3	17.6	22.8	18.7	19.2	19.5	19.3	19.6	22.3
X18:4 ω3	0.0	0.0	18.0	21.9	23.1	23.0	24.6	27.2
X20:0	3.2	0.0	0.0	0.0	0.0	0.0	0.0	0.0
X20:4 ω6	0.0	2.2	1.6	1.5	1.5	1.4	1.3	0.0
X20:5 ω3	0.0	2.9	24.0	26.1	26.0	26.4	26.6	21.4
X22:0	4.8	0.0	0.0	0.0	0.0	0.0	0.0	0.0
X22:2 ω6	0.0	0.0	0.3	0.6	0.8	0.9	0.8	0.7
X22:5 ω6	0.0	0.0	0.0	0.0	0.0	0.0	0.0	3.3
X22:6 ω3	0.0	0.0	0.0	0.2	0.3	0.3	0.4	3.9
X24:0	1.6	0.2	0.0	0.0	0.0	0.0	0.0	0.0
ω3:ω6 ρατιο	3.5	1.6	8.9	10.4	10.8	10.7	11.7	10.2
Μετηοδ	Τοταλ φαττψ αχιδσ							
Πηψτο Χοντριβυτιον		0.00	0.81	0.93	0.96	0.97	1.00	
Μοδελ φιτ (ρ^2)			0.981	0.996	0.999	0.998		
	ω3:ω6 ρατιο							
Πηψτο Χοντριβυτιον		0.00	0.73	0.87	0.92	0.90	1.00	
